# Causal relationships between delirium and Alzheimer's disease: a bidirectional two-sample Mendelian randomization study

**DOI:** 10.1186/s40001-023-01245-w

**Published:** 2023-08-07

**Authors:** Jiang Zheng, Xiaohui Du, Liu Yang, Hong Fu

**Affiliations:** 1 Department of Anesthesiology, Chongqing Emergency Medical Center, Chongqing University Central Hospital, School of Medicine, Chongqing University, Chongqing, China; 2 Department of Neurology, Chongqing Emergency Medical Center, Chongqing University Central Hospital, School of Medicine, Chongqing University, Chongqing, China

**Keywords:** Alzheimer’s disease, Delirium, Mendelian randomization, Causal relationships, European

## Abstract

**Background:**

Previous observational studies have reported that delirium has an association with an increased risk of Alzheimer's disease (AD), and that patients with AD have a higher risk of developing delirium. However, due to the limitations of observational study, it is challenging to confirm whether delirium has a causal effect on AD or reverse causation exists.

**Methods:**

A bidirectional two-sample Mendelian randomization (MR) was performed to investigate the relationship between delirium and AD. Summary statistics from genome-wide association studies of delirium and AD phenotypes were utilized. Inverse-variance weighted (IVW) method was used as the main analysis approach, and additional analyses were performed using MR Egger, weighted median, simple mode and weighted mode to ensure result accuracy. Heterogeneity and horizontal pleiotropy were assessed using Cochran's Q statistics and MR Egger intercept, separately.

**Results:**

The MR analyses showed that genetically predicted delirium was not associated with AD (IVW: odds ratio [OR] 0.98, 95% CI 0.91–1.05, *P* = 0.544; MR Egger: OR 0.98, 95% CI 0.83–1.15, *P* = 0.780; weighted median: OR 0.96, 95% CI 0.88–1.05, *P* = 0.323; simple mode: OR 0.91, 95% CI 0.80–1.04, *P* = 0.212; weighted mode: OR 0.93, 95% CI 0.83–1.05, *P* = 0.277). However, in the reverse direction, AD was associated with delirium (IVW: OR 1.32, 95% CI 1.13–1.54, *P* = 3.91E-04; MR Egger: OR 1.42, 95% CI 1.02–1.98, *P* = 5.60E-02; Weighted median: OR 1.39, 95% CI 1.18–1.63, *P* = 8.22E-05; Simple mode: OR 1.41, 95% CI 1.10–1.80, *P* = 1.41E−02; Weighted mode: OR 1.39, 95% CI 1.16–1.67, *P* = 3.23E-03).

**Conclusion:**

Based on the results of our MR study, there is no bidirectional causality between delirium and AD, delirium is not associated with an increased risk of AD, while genetically predicted AD is a potential causal risk factor for delirium.

**Supplementary Information:**

The online version contains supplementary material available at 10.1186/s40001-023-01245-w.

## Introduction

Delirium is an acute cognitive function statues changing in a short time among hospitalized older adults, which can lead to serious outcomes and increase the medical costs [1, 2]. Delirium is often associated with longer hospital stays, serious complications, and mortality [3–5]. In addition, there is growing evidence suggesting that delirium may also increase the risk of developing dementia. Delirium has been reported to be a strong risk factor for dementia in the patients aged over 85 years and can increase the rate of cognitive deterioration in Alzheimer’s disease (AD) patients [6–8]. However, dementia itself is a significant predisposing factor for delirium, and nearly half of dementia cases go undiagnosed, whether delirium is related to new-onset dementia remains controversial [9–11]. AD is the most common cause of dementia, which accounts of 60–80% of all cases [12]. Although previous observational studies have identified a connection between dementia and delirium, the limitations of those observational studies (i.e., the inability to completely eliminate potential confounding factors, selection bias and small sample size) and the expensive and time-consuming nature of randomized controlled trails, make it challenging to determine a causal association between delirium and AD.

Mendelian randomization (MR) is a valuable tool for exploring causality between exposures and outcomes. By using single nucleotide polymorphisms (SNPs) that linked with exposures, MR can avoid the distribution of confounding bias and reverse causality that are present in observational studies [13, 14]. Therefore, we aimed to investigate the relationship between delirium and AD using a bidirectional two-sample MR in this study.

## Materials and methods

### Study design and MR assumptions

**Fig. 1:**
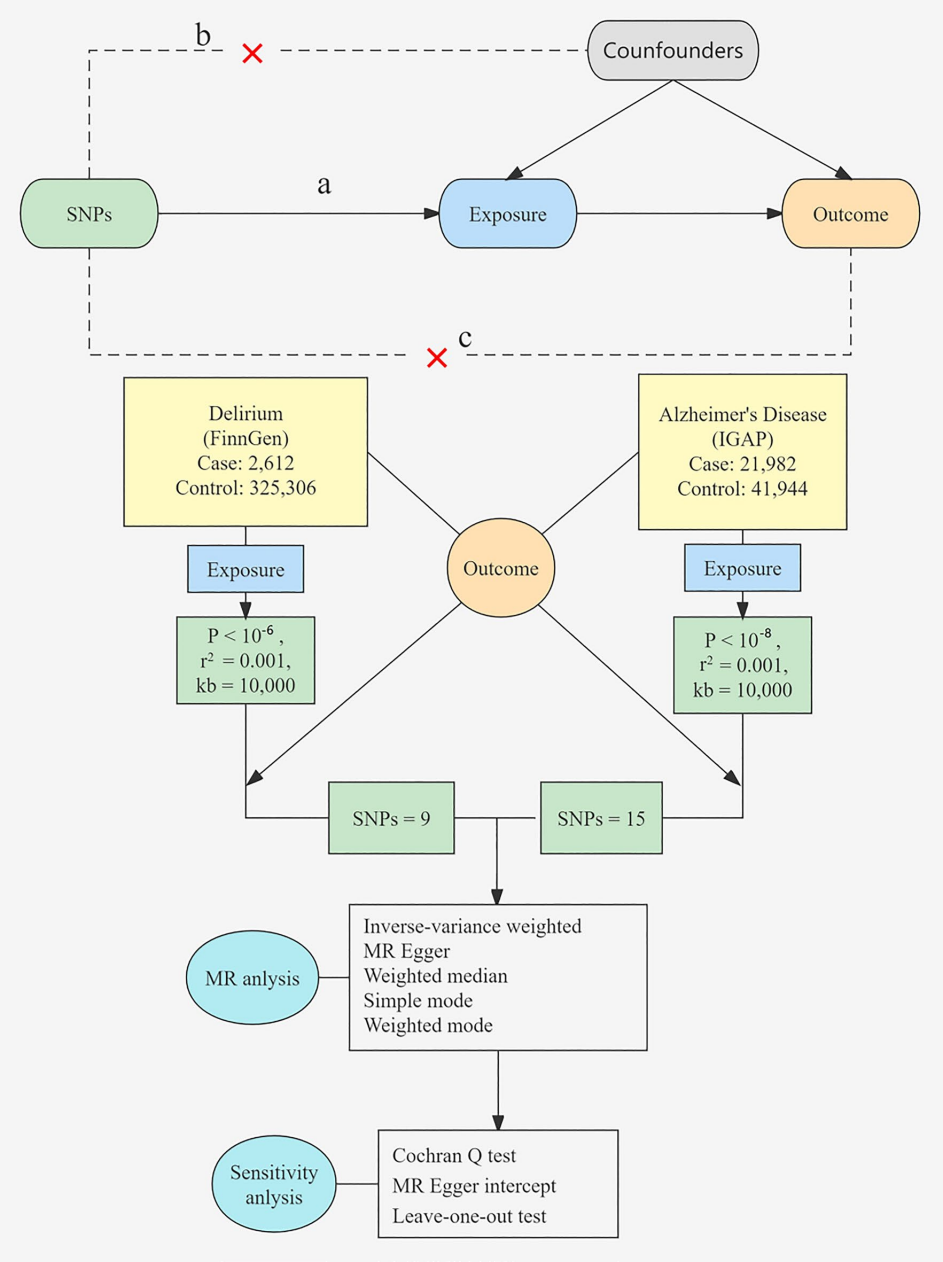
Flowchart of study. Three major assumptions of the MR analysis are as follows: **a** the association hypothesis: genetic variant is associated with exposure; **b** the independence hypothesis: genetic variant is not associated with confounding factors; **c** the exclusivity hypothesis: genetic variables influence the outcome only through the exposure. *IGAP* International Genomics of Alzheimer’s Project, *SNPs* single nucleotide polymorphisms, *MR* Mendelian randomization

Figure 1 presents an overview of the design of our study. The MR analysis rests on following three critical assumptions: (a) the association hypothesis: genetic variant is associated with the exposure; (b) the independence hypothesis: genetic variant is not associated with any confounding factors; and (c) the exclusivity hypothesis: genetic variables only influence the outcome through the exposure.

### Data source

We obtained genome-wide association study (GWAS) summary data related to delirium were sourced from the FinnGen Consortium, based on 2,612 cases and 325,306 controls of Finnish ancestry, and 21,168,109 SNPs were identified. The definition of delirium was based on the International Classification of Diseases, 10th Revision (ICD-10).

Summary data for the GWAS of AD from the International Genomics of Alzheimer’s Project (IGAP) consortium [15]. The GWAS study involved 21,982 patients with AD and 41,944 controls who were non-Hispanic White individuals, and 10,528,610 SNPs were identified.

### Mendelian randomization

SNPs strongly (*P* < 5 × 10^−8^) associated with AD were used as instrumental variables (IVs), and a relaxed threshold (*P* < 5 × 10^−6^) was used to acquire more IVs when exploring the effect of delirium on AD, as a threshold has been used in previous study [16]. For the selection of independent instruments, SNPs that were not in linkage disequilibrium (LD) with each other (*r*^2^ < 0.001) and a distance of 10,000 kb apart were chosen. We then extracted the same SNPs from the outcome GWAS dataset. For removing the ambiguous SNPs, we used the “harmonise_data” function to coordinate exposure and outcome datasets. Each SNP was queried on the website PhenoScanner (http://www.phenoscanner.medschl.cam.ac.uk/), in order to identify and remove potential confounding factors. F-statistic is a valuable tool to assess the strength of the IVs. We calculated the F-statistic for each SNP (*β*^2^/se^2^) and retained SNPs with an F-statistic greater than 10. This threshold was chosen to ensure that the correlation between the IVs and exposure is sufficiently strong to minimize instrumental bias in the MR analysis [17, 18].

We used the random-effect inverse-variance weighted (IVW) method as primary analysis. Multiplicative random-effect IVW model was used when heterogeneity existed, otherwise, a fixed-effect IVW was used [19]. To ensure the accuracy of the results, we used additional methods such ad MR Egger, simple mode, weighted median and weighted mode. As sensitivity analysis, we performed the Cochran Q-test and MR Egger intercept separately to detect any heterogeneity and directional pleiotropy in the IVW model. A significance level of *p* < 0.05 was considered indicative of possible heterogeneity or horizontal pleiotropy. MR Egger can evaluate the pleiotropy using the intercept term, when the intercept term equals zero, the result of MR Egger is consistent with IVW, proving that there is no horizontal pleiotropy [20]. Moreover, a leave-one-out method was used to determine if the combined IVW estimate was driven by any individual SNP.

A bilateral *P*-value < 0.05 was considered statistical significance. We conducted the analysis by the package TwoSampleMR using the R software (Version 4.2.1), and GraphPad Prism for Windows (Version 7.00) was used for visualization.

## Results

### Effect of delirium on Alzheimer’s disease

In the MR analysis, we chose a relaxed threshold (*P* < 5 × 10^−6^) to get more SNPs associated with delirium. Nine SNPs were obtained as IVs after excluding one palindromic SNP (rs963891), one AD-related SNP (rs429558) and two SNPs related to confounding factors (Type II diabetes: rs1353361; major depressive disorder male: rs1669794) [21]. All IVs had F statistics above 10 (rang 21–35), indicating that the strength of the instruments was robust (Additional file 1: Table S1).

**Fig. 2:**
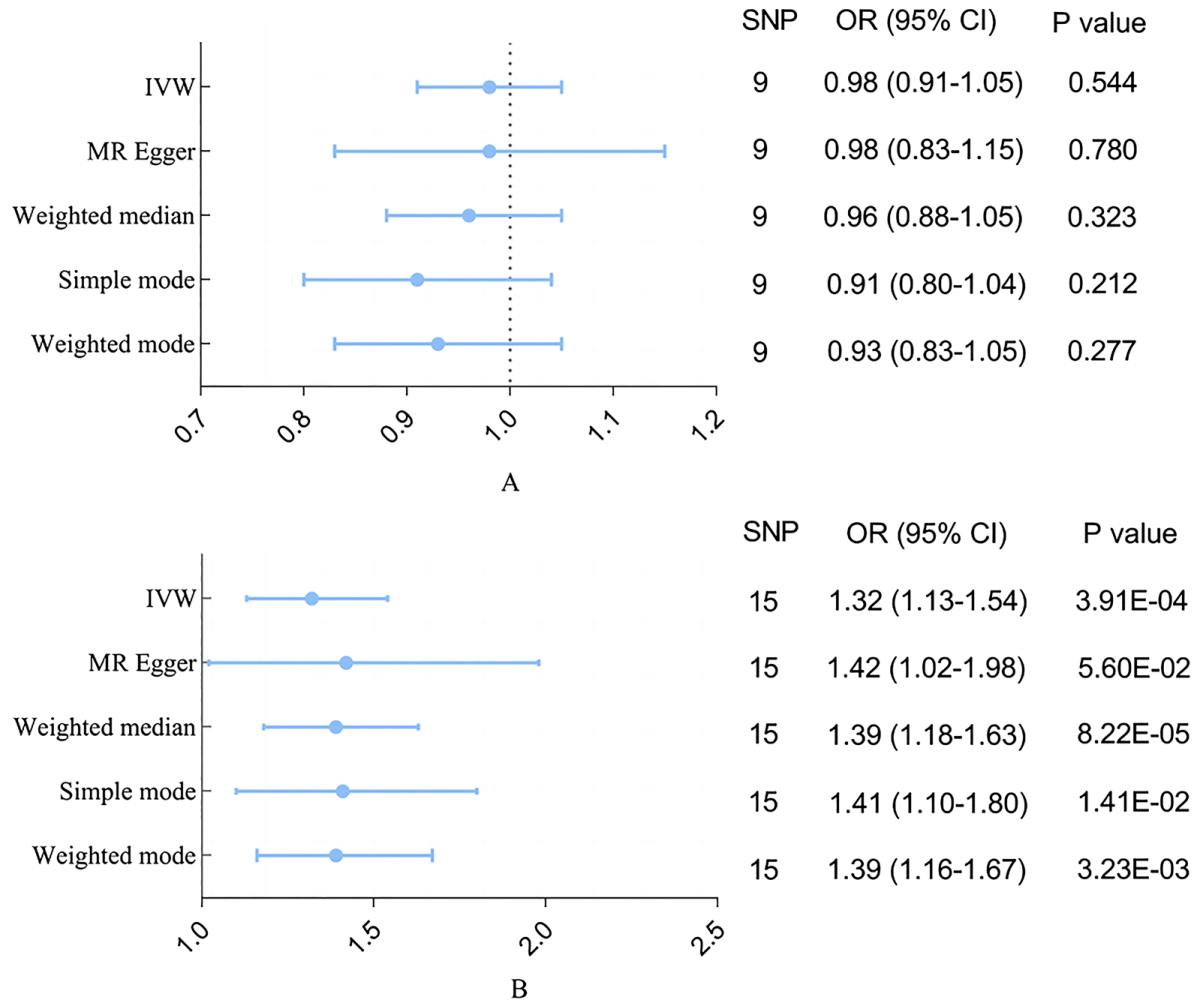
Forest plots of MR estimates for the associations between delirium and AD. **A** Odds ratio plot of delirium on AD using five MR methods. **B** Odds ratio plot of AD on delirium using five MR methods. *AD* Alzheimer's disease, *IVW* inverse-variance weighted, *MR* Mendelian randomization, *OR* odds ratio, *CI* confidence interval, *SNP* single nucleotide polymorphism

**Fig. 3:**
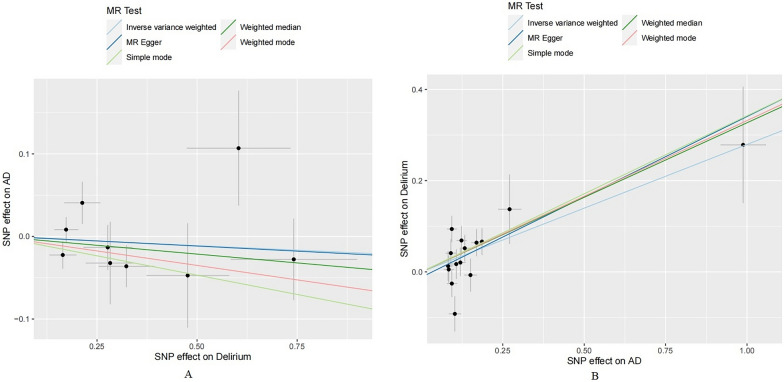
Scatter plot of genetic correlation between AD and delirium using five MR methods. **A** Evaluation the effect of delirium on AD. **B** Evaluation the effect of AD on delirium. *AD* Alzheimer's disease, *MR* Mendelian randomization

**Fig. 4:**
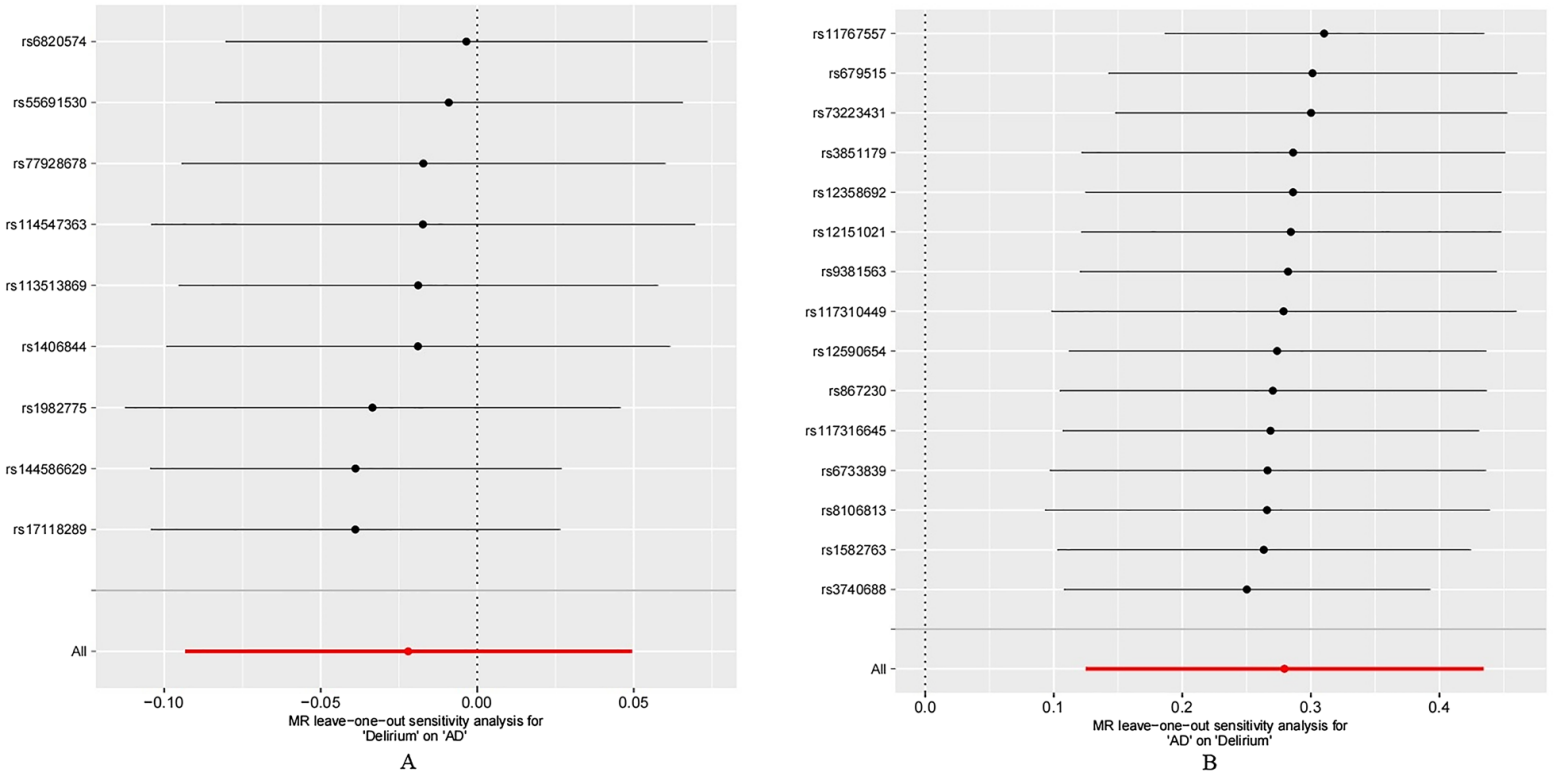
Leave-one-out analysis of the MR results between AD and delirium. **A** Delirium on AD. **B** AD on delirium. *AD* Alzheimer's disease, *MR* Mendelian randomization

Our MR analysis showed that delirium was not associated with AD (IVW: odds ratio [OR] 0.98, 95% CI 0.91–1.05, *P* = 0.544), other MR methods showed a consistent result with IVW (MR Egger: OR 0.98, 95% CI 0.83–1.15, *P* = 0.780; weighted median: OR 0.96, 95% CI 0.88–1.05, *P* = 0.323; simple mode: OR 0.91, 95% CI 0.80–1.04, *P* = 0.212; weighted mode: OR 0.93, 95% CI 0.83–1.05, *P* = 0.277; Figs. 2, 3). There was no evidence of heterogeneity (*Q* = 24.67, *p* = 0.244) and horizontal pleiotropy (*p* = 0.973) (Additional file 1: Table S2). Additionally, the result of leave-one-out sensitivity analysis, also indicated that the causal effect was not driven by a single SNP (Fig. 4).

### Effect of Alzheimer’s disease on delirium

We identified SNPs associated with AD at genome-wide significance level (*P* < 5 × 10^−8^) that were independent from each other (*r*^2^ < 0.001 and kb > 10,000). After excluding palindromic SNPs (rs114812713) and delirium-related SNPs (rs12972156), we obtained 15 SNPs as IVs for our analysis. The F-statistics of all IVs were above 10 (range 30–204), indicating there was no weak instrument (Additional file 1: Table S3).

Figure 2 illustrates the results of the MR analysis. All four methods except MR Egger showed a statistical significance (IVW: OR 1.32, 95% CI 1.13–1.54, *P* = 3.91E−04). Weighted median, simple mode and weighted mode methods all show a consistent result with IVW (weighted median: OR 1.39, 95% CI 01.18–1.63, *P* = 8.22E−05; simple mode: OR 1.41, 95% CI 1.10–1.80, *P* = 1.41E−02; weighted mode: OR 1.39, 95% CI 1.16–1.67, *P* = 3.23E−03). Although MR Egger did not show an association in AD and delirium, it was close to statistical significance (OR 1.42, 95% CI 1.02–1.98, *P* = 5.70E-02) (Figs. 2, 3). Heterogeneity was observed in the Cochran’s Q test (*Q* = 24.67, *p* = 0.029). No horizontal pleiotropy was observed in the MR Egger intercept test (*p* = 0.630), indicating that the associations were not confounded by pleiotropic effects (Additional file 1: Table S2). MR leave-one-out sensitivity analysis suggested that no single SNP changed the causal effect (Fig. 4).

## Discussion

We conducted a bidirectional two-sample MR approach to evaluate the causal association between delirium and AD. Results from IVW and other MR methods, showed that there was no bidirectional causality between delirium and AD. Specifically, we found that patients with AD may have a higher risk of delirium, but delirium was not significant in relation to AD. To the best of our knowledge, the present study firstly explored a causal relationship between delirium and AD using MR. Our findings provide valuable insights into the potential causal relationship between these two conditions.

Previous studies have found a correlation between delirium and AD. Delirium has been identified as a risk factor that increases the likelihood of developing or exacerbating dementia, and it can accelerate cognitive decline in AD patients [6, 22, 23]. However, it is important to note that in the present study, we only included patients with or without AD, and our research focused on assessing the causal relationship between delirium and AD. The process and development of AD go beyond the scope of this specific study. Although we did not find that delirium can increase the risk for developing AD, it is not contradictory to the conclusion that delirium can accelerate cognitive decline in AD patients. Inflammatory response is believed to be a primary driver of acute brain dysfunction or delirium, in addition, critical illness with acute inflammatory insult was also a risk factor for AD [24, 25]. A meta-analysis including 28 observational studies found that biomarkers associated with dementia, such as elevated blood-based levels of IL-6, C-reactive protein, S100B, and NfL, were also associated with delirium [26]. The fact that delirium and AD share similar biomarkers suggests that they may share similar pathologies in the development of disease. These biomarkers associated delirium may also cause harm to the process of AD and speed up cognitive decline instead of delirium itself.

There are common risk factors, such as low vitamin D concentrations, that may contribute to both delirium and AD. Two MR analysis studies have found an association between low vitamin D concentrations and the development of AD and delirium [27, 28]. Witlox J et al. concluded that patients with delirium have a higher incidence of developing AD in a meta-analysis. This meta-analysis included two prospective follow-up studies that were conducted over a period of 5 and 3 years [5, 29, 30]. However, causality cannot be established because of the small sample size and the limitation of observational studies. In this study, we used five MR analysis methods and found consistent results showing that delirium is not an independent risk factor for AD. The results were robust, as we did not observe any pleiotropy or heterogeneity in the sensitivity analysis. For all that, we cannot completely deny the possibility that delirium may influence the process of AD through similar pathological mechanisms, such as abnormal nerve transmission and neuroinflammation. Future studies including lager sample sizes should be done, to confirm whether delirium has an effect on the process of AD and to explore the true underlying factors that contribute to accelerating the process of AD.

It is well known that AD is the risk factors for delirium. In our study, the effect estimate of IVW (OR 1.32, 95% CI 1.13–1.54) demonstrates that AD is indeed a risk factor for delirium, which aligns with previous research demonstrating that dementia and recognition decline are independent risk factors for delirium [31, 32]. Although the exact mechanisms of delirium are not completely understood, it is believed to be a multifactorial process that can result in brain dysfunction, often involving abnormal nerve transmission and neuroinflammation [10, 33, 34]. Patients with dementia and pre-existing neurodegeneration may be more prone to neuroinflammatory cytokines, and trigger greater oxidative stress during surgery, pain or other stressful situations, potentially increasing the risk of delirium. Furthermore, patients with delirium superimposed on dementia have poorer outcomes, such as higher mortality rates, greater dependence on walking and increased healthcare expenses [35–37]. Therefore, recognition and prevention of the occurrence of delirium at an early stage is crucial for improving patient outcomes.

Delirium is currently classified into major subtypes: hyperactive type (which is more apparent), hypoactive type (which is more prevalent but often overlooked) and a combined hyperactive and hypoactive types [38]. Research has found that staff and volunteers tend to focus more on treating hyperactive symptoms, while may miss the prevention and early recognition of hypoactive delirium [39]. Clinicians and nurses may overlook patients with hypoactive delirium because they appear to be performing normally, even though they actually unwell. Prevention is always better than treatment, and there are two types of risk factors for delirium: predisposing and precipitating factors. The predisposing factors are associated with the patients’ internal state, such as advanced age, functional deficiency, and comorbidities. Precipitating factors, on the other hand, refer to external disturbances, such a trauma, sedative drugs, sepsis, surgery and anesthesia. Patients with more predisposing factors more likely to develop delirium with fewer precipitating factors [2, 40]. Post-operative delirium is a common post-surgical complication, particularly in older adults [41]. Although it may be difficult to make changes in predisposing factors, it possible to mitigate the risk of precipitating factors. Clinicians can avoid the use of benzodiazepines or anticholinergic drugs, manage pain, encourage early post-operative mobilization and regular ambulation to reduce the risk of post-operative delirium. Implementation nonpharmacologic prevention strategies can also significantly improve outcomes for older patients [42]. Therefore, the clinicians and nurses should pay close attention to patients with AD, and the presence of predisposing risk factors for delirium. By taking early preventative measures, clinicians can improve the chances of early recognition and effective prevention of delirium.

Several limitations of our study should be noted. Firstly, our findings may not be applicable to populations with different ethnicities or geographic locations as our study only included individuals of European ancestry. Further data collection and analysis is needed to confirm the generalizability of our findings. Secondly, we only included the delirium phenotype as a binary variable, and did not consider the three subtypes or delirium or the severity of delirium. Therefore, we could not assess the association between AD and delirium subtypes or severity. It is worth noting that previous study has shown that patients with persistent delirium have worse outcomes than those with transient delirium [43]. Thirdly, the Cochran’s Q test revealed heterogeneity in the effect of AD on delirium. Various GWAS cohort studies might have differences in gene annotation analysis platforms, inclusion and exclusion criteria for cases, which could contribute to the heterogeneity in our study [44]. Additionally, the GWAS data for AD are obtained from several consortia, may contribute to the heterogeneity. However, this heterogeneity did not impact the main analysis, as the consistent results from other MR methods, such as MR Egger and weighted median model, are robust to genetic heterogeneity and can provide valid estimates [45–47]. Moreover, the other three MR methods (simple mode, weighted median and weighted mode) showed similar result (*p* < 0.05), and the p value was also close to statistical significance using MR Egger method (*P* = 5.70E−02). In addition, no directional pleiotropy was observed in the MR Egger intercept analysis. The leave-one-out sensitivity analysis was also performed, and it showed that even when individual SNPs were removed one by one, the overall results remained stable (the overall error line did not fluctuate significantly). These findings strongly suggest that the results of our MR analysis are robust.

## Conclusions

Our bidirectional two-sample MR analysis showed that although AD increase the risk for delirium, while delirium is not associated with AD. These findings may suggest that clinicians should identify and prevent the occurrence of delirium superimposed on dementia in an early stage. Future study should investigate the mechanisms underlying the effect of AD on delirium, and researchers could include larger sample sizes to confirm the effect of delirium on AD.

### Supplementary Information

**Additional file 1: Table S1.** SNPs of delirium. **Table S2.** Heterogeneity and Horizontal pleiotropy analysis of IVW between AD and delirium. **Table S3.** SNPs of AD.

## Data Availability

The GWAS summary data for AD (ieu-b-2) and Delirium (finngen-R8-F5_DELIRIUM) are publicly available, and can be downloaded from GWAS (https://gwas.mrcieu.ac.uk/) and FinnGen website (https://www.finngen.fi/en).

## Abbreviations

AD: Alzheimer’s disease
MR: Mendelian randomization
IVW: Inverse-variance weighted
SNPs: Single nucleotide polymorphisms
GWAS: Genome-wide association study
IGAP: International Genomics of Alzheimer’s Project
LD: Linkage disequilibrium
OR: Odds ratio
