# Supplementary material for: Causal relationships between delirium and Alzheimer's disease: a bidirectional two-sample Mendelian randomization study
Source: Eur J Med Res. 2023 Aug 7;28:271. doi: 10.1186/s40001-023-01245-w (PMC10405368; doi:10.1186/s40001-023-01245-w)
Supplement: Supplementary file 1 — Additional file 1: Table S1. SNPs of delirium. Table S2. Heterogeneity and Horizontal pleiotropy analysis of IVW between AD and delirium. Table S3. SNPs of AD. [file 40001_2023_1245_MOESM1_ESM.docx]

**Supplementary Material**

**Causal Relationships Between** **Alzheimer's Disease and Delirium: A Bidirectional Two-Sample Mendelian Randomization Study**

Jiang Zheng ^1,†^, Xiaohui Du ^1,†^ Liu Yang ^2^, Hong Fu ^1,*^

***Correspondence:** Hong Fu: fuhong1974@cqu.edu.cn

1. Table S1: SNPs of delirium

| SNP | Effect_allele | Other_allele | Beta | SE | P value | F |
| --- | --- | --- | --- | --- | --- | --- |
| rs113513869 | A | G | 0.282882 | 0.060899 | 3.40E-06 | 22 |
| rs114547363 | T | G | 0.7414 | 0.157376 | 2.46E-06 | 22 |
| rs1406844 | T | C | 0.276813 | 0.0605737 | 4.88E-06 | 21 |
| rs144586629 | G | A | 0.603589 | 0.128977 | 2.87E-06 | 22 |
| rs17118289 | G | A | 0.213211 | 0.0445867 | 1.74E-06 | 23 |
| rs1982775 | T | C | 0.172858 | 0.0293104 | 3.69E-09 | 35 |
| rs55691530 | A | G | 0.164516 | 0.033101 | 6.69E-07 | 25 |
| rs6820574 | G | T | -0.323264 | 0.068918 | 2.72E-06 | 22 |
| rs77928678 | G | A | -0.476477 | 0.102655 | 3.46E-06 | 22 |

Abbreviations: SNP, single nucleotide polymorphism; SE, standard error.

1. Table S2. Heterogeneity and Horizontal pleiotropy analysis of IVW between AD and delirium

|  |  | Heterogeneity | | Horizontal pleiotropy | |
| --- | --- | --- | --- | --- | --- |
| Exposure | Outcome | Cochran's Q | P value | Intercept | P value |
| AD | Delirium | 24.67 | 0.029 | -0.011 | 0.630 |
| Delirium | AD | 10.30 | 0.244 | 0.0008 | 0.973 |

Abbreviations: IVW, Inverse-variance weighted; AD, Alzheimer's disease.

1. Table S3: SNPs of AD

| SNP | Effect_allele | Other_allele | Beta | SE | P value | F |
| --- | --- | --- | --- | --- | --- | --- |
| rs117310449 | T | C | 0.9879 | 0.0691 | 2.30E-46 | 204 |
| rs117316645 | A | G | 0.2709 | 0.0349 | 8.35E-15 | 60 |
| rs11767557 | C | T | -0.1028 | 0.0182 | 1.62E-08 | 32 |
| rs12151021 | G | A | -0.1071 | 0.0169 | 2.34E-10 | 40 |
| rs12358692 | T | C | 0.0841 | 0.0154 | 4.73E-08 | 30 |
| rs12590654 | A | G | -0.0906 | 0.0157 | 7.89E-09 | 33 |
| rs1582763 | A | G | -0.1232 | 0.0149 | 1.36E-16 | 68 |
| rs3740688 | T | G | 0.0935 | 0.0144 | 8.41E-11 | 42 |
| rs3851179 | C | T | 0.1198 | 0.0148 | 5.75E-16 | 66 |
| rs6733839 | T | C | 0.1693 | 0.0154 | 4.11E-28 | 121 |
| rs679515 | C | T | -0.1508 | 0.0183 | 1.72E-16 | 68 |
| rs73223431 | T | C | 0.0936 | 0.0153 | 9.50E-10 | 37 |
| rs8106813 | G | A | -0.1861 | 0.021 | 7.87E-19 | 79 |
| rs867230 | A | C | 0.1333 | 0.0158 | 3.26E-17 | 70 |
| rs9381563 | T | C | -0.0821 | 0.0148 | 2.90E-08 | 31 |

Abbreviations: AD, Alzheimer's disease; SNP, single nucleotide polymorphism; SE, standard error.
